# epiCaPture: A Urine DNA Methylation Test for Early Detection of Aggressive Prostate Cancer

**DOI:** 10.1200/PO.18.00134

**Published:** 2019-01-14

**Authors:** Eve O’Reilly, Alexandra V. Tuzova, Anna L. Walsh, Niamh M. Russell, Odharnaith O’Brien, Sarah Kelly, Odharna Ni Dhomhnallain, Liam DeBarra, Connie M. Dale, Rick Brugman, Gavin Clarke, Olivia Schmidt, Shane O’Meachair, Dattatraya Patil, Kathryn L. Pellegrini, Neil Fleshner, Julia Garcia, Fang Zhao, Stephen Finn, Robert Mills, Marcelino Y. Hanna, Rachel Hurst, Elizabeth McEvoy, William M. Gallagher, Rustom P. Manecksha, Colin S. Cooper, Daniel S. Brewer, Bharati Bapat, Martin G. Sanda, Jeremy Clark, Antoinette S. Perry

**Affiliations:** **Eve O’Reilly**, **Alexandra V. Tuzova**, **Niamh M. Russell**, **Gavin Clarke**, **Olivia Schmidt**, **William M. Gallagher**, and **Antoinette S. Perry**, University College Dublin; **Eve O’Reilly**, **Alexandra V. Tuzova**, **Anna L. Walsh**, **Sarah Kelly**, **Odharna Ni Dhomhnallain**, **Liam DeBarra**, **Connie M. Dale**, **Rick Brugman**, **Shane O’Meachair**, **Rustom P. Manecksha**, and **Antoinette S. Perry**, Trinity College Dublin; **Anna L. Walsh**, **Odharnaith O’Brien**, **Stephen Finn**, and **Rustom P. Manecksha**, St James’s Hospital; **Elizabeth McEvoy** and **Rustom P. Manecksha**, Tallaght Hospital, Dublin, Ireland; **Dattatraya Patil**, **Kathryn L. Pellegrini**, and **Martin G. Sanda**, Emory University School of Medicine, Atlanta, GA; **Neil Fleshner**, **Julia Garcia**, **Fang Zhao**, and **Bharati Bapat**, University of Toronto, Toronto, Ontario, Canada; **Robert Mills**, Norfolk and Norwich University Hospital; **Marcelino Y. Hanna**, **Rachel Hurst**, **Colin S. Cooper**, **Daniel S. Brewer**, and **Jeremy Clark**, University of East Anglia; and **Daniel S. Brewer**, Earlham Institute, Norwich, England.

## Abstract

**Purpose:**

Liquid biopsies that noninvasively detect molecular correlates of aggressive prostate cancer (PCa) could be used to triage patients, reducing the burdens of unnecessary invasive prostate biopsy and enabling early detection of high-risk disease. DNA hypermethylation is among the earliest and most frequent aberrations in PCa. We investigated the accuracy of a six-gene DNA methylation panel (Epigenetic Cancer of the Prostate Test in Urine [epiCaPture]) at detecting PCa, high-grade (Gleason score greater than or equal to 8) and high-risk (D’Amico and Cancer of the Prostate Risk Assessment] PCa from urine.

**Patients and Methods:**

Prognostic utility of epiCaPture genes was first validated in two independent prostate tissue cohorts. epiCaPture was assessed in a multicenter prospective study of 463 men undergoing prostate biopsy. epiCaPture was performed by quantitative methylation-specific polymerase chain reaction in DNA isolated from prebiopsy urine sediments and evaluated by receiver operating characteristic and decision curves (clinical benefit). The epiCaPture score was developed and validated on a two thirds training set to one third test set.

**Results:**

Higher methylation of epiCaPture genes was significantly associated with increasing aggressiveness in PCa tissues. In urine, area under the receiver operating characteristic curve was 0.64, 0.86, and 0.83 for detecting PCa, high-grade PCa, and high-risk PCa, respectively. Decision curves revealed a net benefit across relevant threshold probabilities. Independent analysis of two epiCaPture genes in the same clinical cohort provided analytical validation. Parallel epiCaPture analysis in urine and matched biopsy cores showed added value of a liquid biopsy.

**Conclusion:**

epiCaPture is a urine DNA methylation test for high-risk PCa. Its tumor specificity out-performs that of prostate-specific antigen (greater than 3 ng/mL). Used as an adjunct to prostate-specific antigen, epiCaPture could aid patient stratification to determine need for biopsy.

## INTRODUCTION

Prostate cancer (PCa) is the most common noncutaneous malignancy in men and the third leading cause of cancer-related deaths in men in the Western world. In 2012, there were an estimated 1.1 million men diagnosed with this disease. With an aging population, spread of Westernized diet, and increasing use of prostate-specific antigen (PSA) testing, this figure is predicted to double by 2035.^1^

PCa demonstrates extreme clinical heterogeneity. Overall, 10-year survival rates are close to 100%^2^; thus, there is much interest in strategies to reduce overtreatment of low/intermediate-risk tumors.^3^ However, more than 300,000 men die as a result of PCa each year. This stark figure, together with the knowledge that overtreatment comes with the high price of prevalent life-changing side effects, illustrates the need for tools to selectively identify high-risk PCa (those tumors with a high propensity to metastasize) at an early stage, while the disease is potentially curable.

Research is ongoing into methods to address this need and better risk-stratify patients. Developments include algorithms around PSA isoforms (ie, the four-kallikrein and Prostate Health Index tests),^4^ and multifactorial risk-calculator nomograms, such as the Prostate Cancer Prevention Trial and European Randomized Study of Screening for Prostate Cancer.^5^ Urine in particular is an attractive noninvasive route, with the potential to reduce the number of patients subjected to prostate biopsy. Urinary analysis of molecular correlates of aggressive disease could be used to triage patients and identify those who actually require an invasive prostate biopsy to histologically diagnose and grade their disease.

In 2012, Movember launched four collaborative Global Action Plan (GAP) initiatives, focusing on PCa biomarkers in urine, plasma, serum, and exosomes. The aim of the GAP1 urine project was to develop multiparametric urine biomarkers to facilitate early detection of aggressive PCa. Underpinning this aim was the cooperative establishment of prebiopsy post–digital rectal examination (DRE) urine biorepositories at each institution, with a confederated database housing accompanying clinical, pathologic, and lifestyle data. The GAP1 urine project united investigators from seven different countries studying proteomic, transcriptomic, and epigenetic analytes.

Herein, we present the results of one of two DNA methylation panels investigated in GAP1 urine, adhering to the REMARK (Reporting Recommendations for Tumor Markers) guidelines on correct reporting of prognostic biomarker research.^6^ The Epigenetic Cancer of the Prostate Test in Urine (epiCaPture) is a multibiomarker panel that quantitatively measures DNA hypermethylation at the 5′-regulatory regions of six genes (*GSTP1*, *SFRP2*, *IGFBP3*, *IGFBP7*, *APC*, and *PTGS2*), all of which have previously been reported in PCa.^7-10^ Cumulative evidence supports promoter hypermethylation and concomitant gene silencing in PCa initiation^7,11^ and as a strong prognostic indicator.^12,13^ The purpose of this study was to determine the accuracy of epiCaPture for noninvasive detection of PCa and aggressive PCa, as defined by Gleason score (greater than or equal to 8) on biopsy, as well as two widely used risk-stratification systems, namely, D’Amico^14^ and Cancer of the Prostate Risk Assessment (CAPRA).^15^

## PATIENTS AND METHODS

### Patient Population

**Fig 1.**
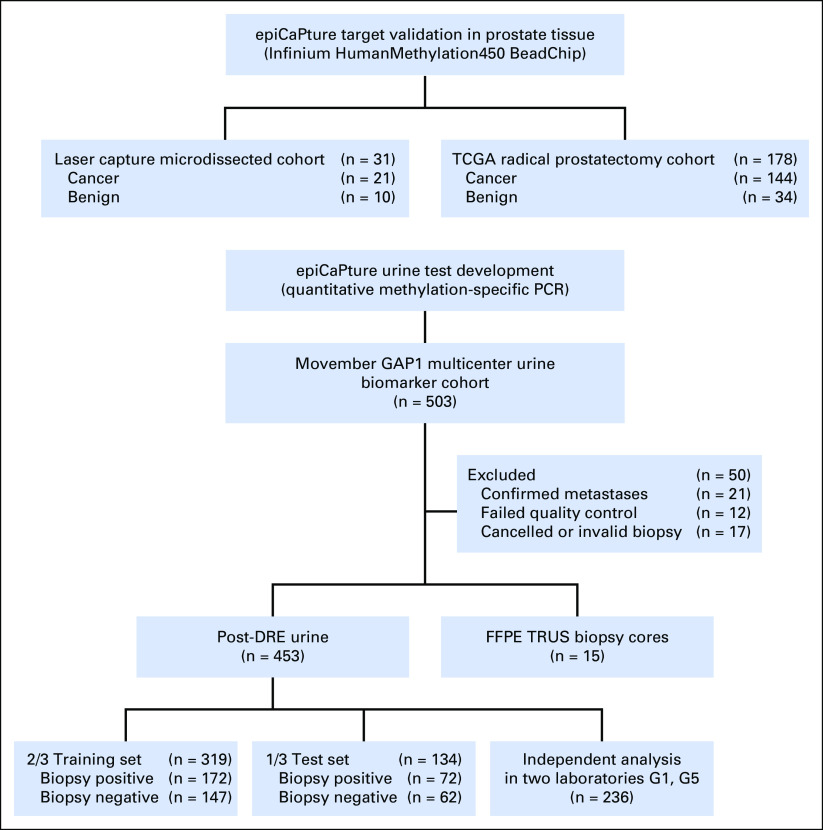
Flow diagram of specimens used in the study. DRE, digital rectal examination; epiCaPture, Epigenetic Cancer of the Prostate Test in Urine; FFPE, formalin-fixed paraffin-embedded; GAP, Global Action Plan; PCR, polymerase chain reaction; TCGA, The Cancer Genome Atlas; TRUS, transrectal ultrasound.

This institutional review board–approved, multicenter prospective study enrolled men who were scheduled for transrectal ultrasound-guided (TRUS) biopsy because of an elevated and/or increasing PSA and/or a suspicious DRE. Six centers from the United States (Emory Healthcare, Atlanta, GA), Canada (University Health Network and Lunenfeld-Tanenbaum Research Institute, Sinai Health Systems, Toronto), and Europe (St James’s Hospital, Mater Misericordiae University Hospital, and Tallaght Hospital, all in Ireland; Norfolk and Norwich University Hospital, England), collected prebiopsy, post-DRE urine (≤ 50 mL) between January 2012 and October 2015. Exclusion criteria included previous diagnosis of PCa, active urinary tract infection, recent prostate biopsy or transurethral resection of the prostate (less than 6 wk), and confirmed presence of metastatic disease (by pelvic magnetic resonance imaging or bone scan). Histopathologic evaluation of TRUS biopsy cores was carried out at each site as per standard procedure by local consultant histopathologists. In total, urine samples were collected from 503 men, with matched formalin-fixed paraffin-embedded TRUS biopsy cores acquired, where available (Fig 1). Signed informed consent was obtained from all patients. The demographic, clinical, and pathologic data for the whole cohort are listed in Table 1. In addition to this prebiopsy population, a retrospective analysis of the epiCaPture gene loci was performed on two independent cohorts of prostate tissues (Fig A2; Data Supplement).

**Table 1.**
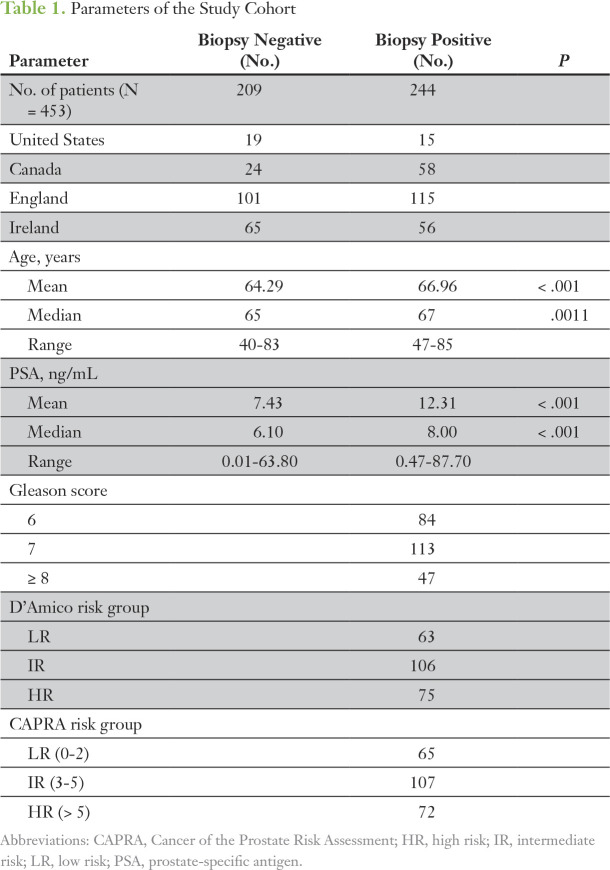
Parameters of the Study Cohort

### Sample Collection, Processing, and epiCaPture Analysis

Urine samples were stored on ice and processed within 4 hours of collection, following a Movember GAP1 standard operating procedure. Details of nucleic acid isolation and quantification and epiCaPture analysis are provided in Appendix Table A1 and the Data Supplement.

### Statistical Analysis

Statistical analyses were performed with R version 3.4.1 and GraphPad Prism version 6. Two-tailed tests were used, and *P* < .05 was considered significant. The ability of epiCaPture genes (or the combination thereof), when applied to urine, to predict cancer (*v* no cancer detected on biopsy), high-grade cancer (Gleason score greater than or equal to 8 *v* all else), and high-risk cancer (*v* all else) as classified by D’Amico and CAPRA was assessed by logistic regression models, built in a two thirds training set (n = 319) and validated in a one third test set (n = 134). Discriminatory ability of the models was assessed via sensitivity, specificity, positive predictive value, and negative predictive value (NPV) using the Youden index. Diagnostic potential was quantified using the area under the receiver operating characteristic curve (AUC). Clinical utility was assessed by decision curve analysis (DCA).^16^

## RESULTS

### epiCaPture Genes Demonstrate Prognostic Utility in Prostate Tissues

**Fig 2.**
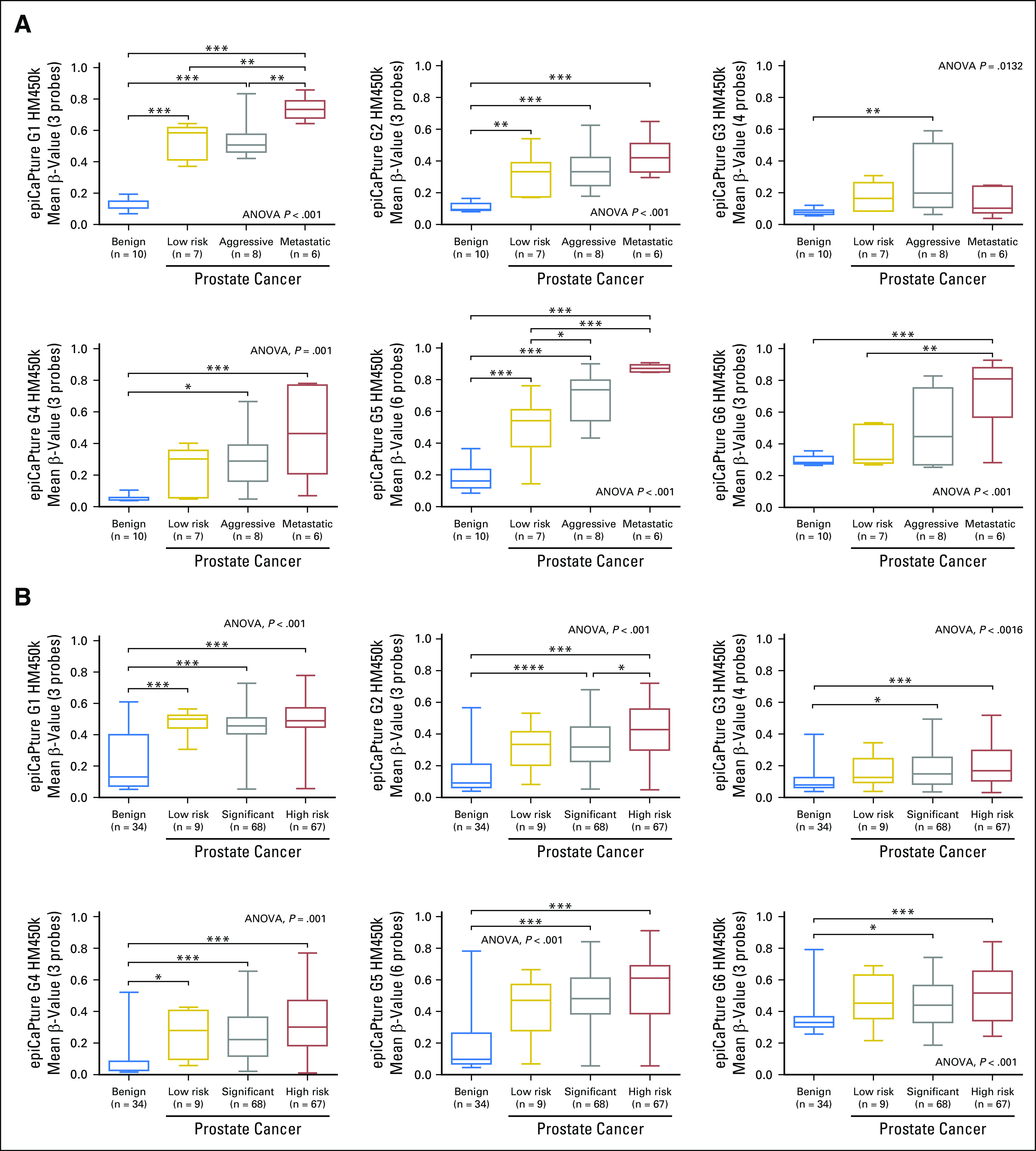
Epigenetic Cancer of the Prostate Test in Urine (epiCaPture) gene validation in prostate tissue specimens. DNA methylation was measured using the Infinium HumanMethylation450 BeadChip (HM450k) in two independent cohorts of patients undergoing radical prostatectomy. (A) Laser capture microdissected enriched prostate epithelial cells from benign prostate tissue (procured from men undergoing radical cystoprostatectomy for bladder cancer, with no clinical or histologic evidence of prostate cancer [PCa]) and low-risk, aggressive, and metastatic PCa. (B) Data extracted from the TCGA repository for matched-benign prostate tissue and low-risk, significant, and high-risk PCa, all procured from men undergoing radical prostatectomy. A full description of the two cohorts is provided in the Data Supplement. Each data set was analyzed by one-way analysis of variance (ANOVA), followed by post hoc analyses using the Tukey-Kramer multiple comparisons test. Boxes indicate the 25th and 75th percentiles, the line represents the median, and the whiskers show the minimum and maximum value in each cohort of samples. Significance is indicated by *P < .05, **P < .01, and *** P < .001, and **** P < .0001

Mindful that most potential biomarkers fail to translate to the clinic,^17^ we first set out to verify the prognostic utility of each epiCaPture constituent gene by measuring its methylation in two independent cohorts of radical prostatectomy samples. There was strong evidence for differences in mean methylation β-values between four different groups (benign, low-risk, aggressive, and metastatic PCa) in a small cohort of prostate tissues (subjected to epithelial cell enrichment) measured using the Infinium HumanMethylation450 BeadChip (Fig 2A). Post hoc analyses revealed significantly higher methylation of each gene in aggressive and/or metastatic lesions compared with benign tissue. Extracting freely available data from the same analytical platform for a larger The Cancer Genome Atlas cohort yielded similar results; each epiCaPture gene demonstrated significantly higher levels of DNA methylation in clinically significant and high-risk tumors, relative to histologically benign tissue (Fig 2B). Although there was some evidence of methylation in low-risk disease, only G1 (*GSTP1*) was methylated at significantly higher levels than benign tissue in both cohorts.

### epiCaPture Performance Characteristics in Urine

**Fig 3.**
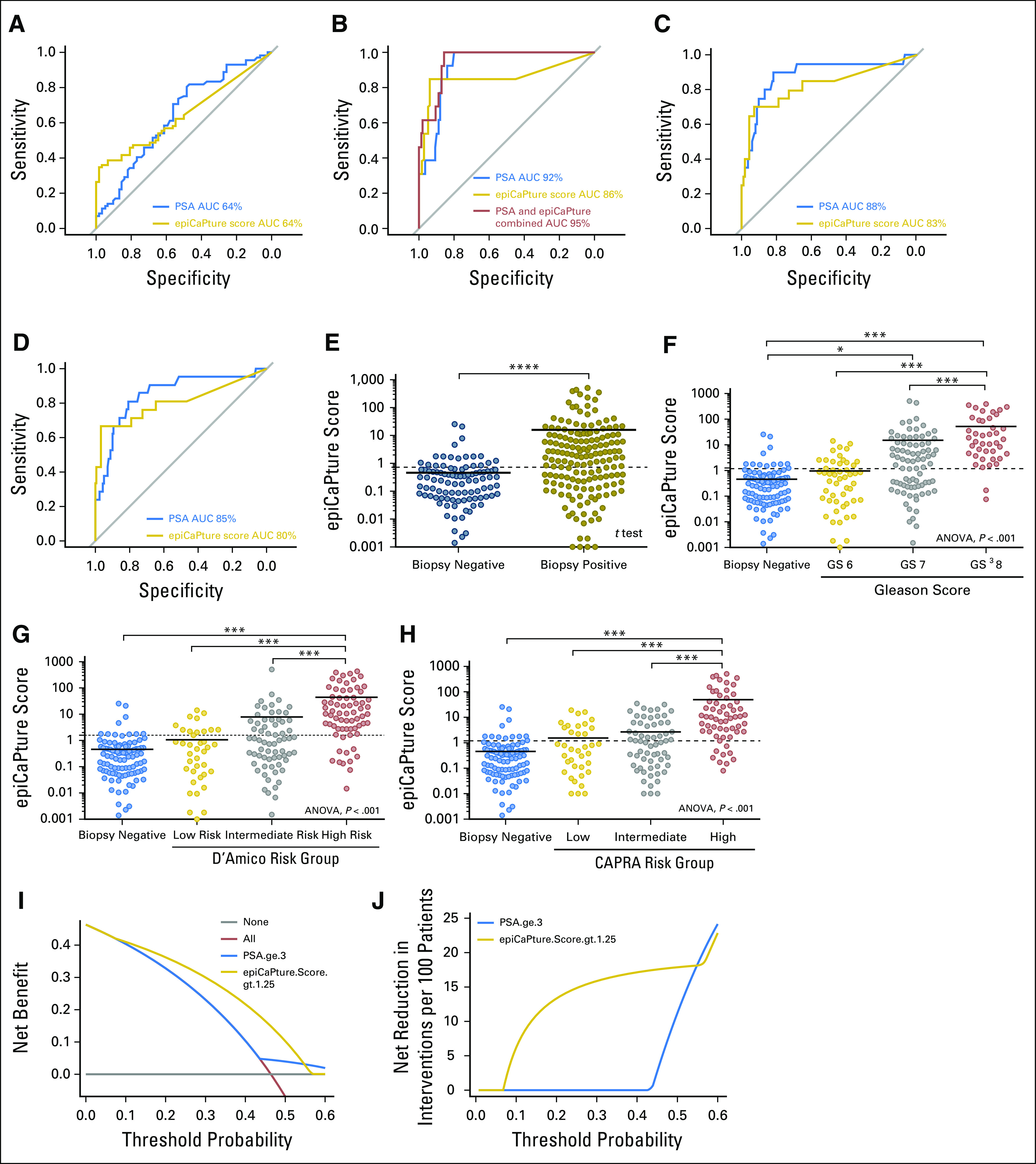
Epigenetic Cancer of the Prostate Test in Urine (epiCaPture) performance at noninvasive detection of prostate cancer in urine. The performance of epiCaPture compared with/in conjunction with prostate-specific antigen (PSA; treated as a continuous variable) in the test set (n = 134) for predicting each end point was assessed by receiver operating characteristic curves: (A) cancer (*v* no cancer detected on transrectal ultrasound biopsy), (B) high-grade cancer (Gleason score greater than or equal to 8 *v* all else), (C) D’Amico high-risk cancer (*v* all else), and (D) CAPRA (Cancer of the Prostate Risk Assesment) high-risk cancer (*v* all else). The distribution of epiCaPture scores in the whole study population (n = 453), viewed by (E) biopsy outcome, (F) Gleason grade, (G) risk categorization by D’Amico risk-classification systems, and (H) risk categorization by CAPRA risk-classification system. The epiCaPture score was calculated for each sample by summing the normalized index of methylation (Data Supplement) for G1 to G6. epiCaPture score distributions were analyzed by *t* test or one-way analysis of variance (ANOVA), followed by post hoc analyses using the Tukey-Kramer multiple comparisons test. For ease of interpretation, epiCaPture scores are plotted on a logarithmic axis, and samples that were negative (epiCaPture score = 0) are not shown. Solid black line represents the mean of each group. Dashed lines indicate the epiCaPture score thresholds derived in the training set to maximize both sensitivity and specificity. Significance is indicated by *P < .05, **P < .01, ***P < .001, and ****P < .0001. (I) Decision curve analysis demonstrated net clinical benefit of performing biopsy on patients in the test set on the basis of an epiCaPture score greater than 1.25 versus PSA greater than or equal to 3 ng/mL across a range of clinically relevant threshold probabilities (the risk of cancer, such that a patient would choose to undergo biopsy by weighing the relative harms of false-positive and false-negative predictions). The horizontal gray line represents the decision curve for the performing biopsy on no patients and the red line represents the decision curve for performing biopsy on all patients. (J) This translates to a net reduction in biopsies of approximately 15%, as compared with PSA, across a range of threshold probabilities. AUC, area under the receiver operating characteristic curve.

**Fig A1.**
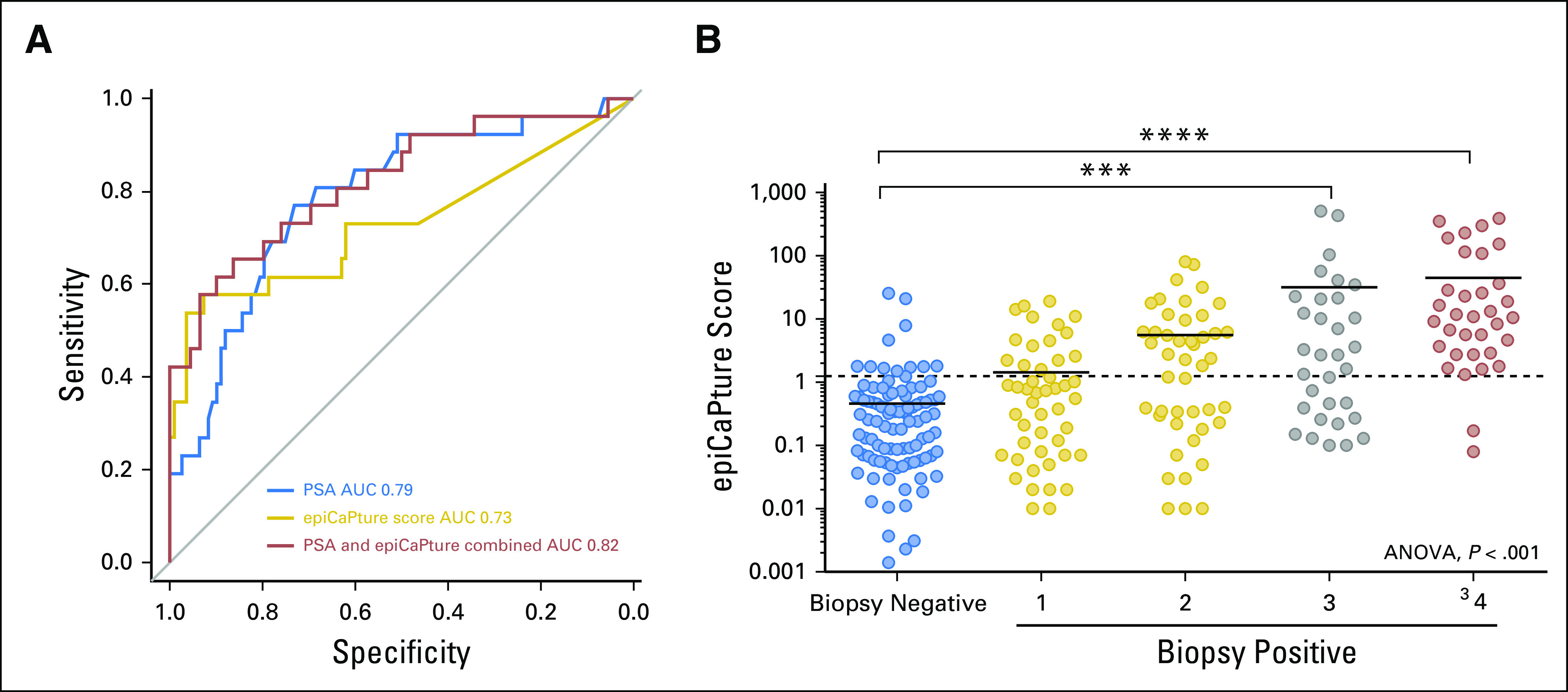
(A) The performance of epiCaPture compared to and in conjunction with prostate-specific antigen (PSA; treated as a continuous variable) in the test set (n = 134) for predicting ISUP group 3, 4 and 5 PCa on biopsy was assessed by receiver operator characteristic curves. (B) The distribution of epiCaPture scores in biopsy-negative (no cancer detected) *v* biopsy-positive patients, categorized by ISUP group, shows increasing methylation by group. Significance is indicated by ***P < .001 and ****P < .0001. ANOVA, analysis of variance; AUC, area under the receiver operating curve.

In total, epiCaPture was performed on 453 urine samples (Fig 3). Logistic regression models were developed using a 319-specimen training set (corresponding to 70% of the total cohort) and then evaluated for their ability to correctly predict cancer/high-grade cancer/high-risk cancer in the remaining test set. Analyzing all six genes together (epiCaPture score) consistently performed better than individual genes or combinations thereof. epiCaPture showed a comparable ability to predict cancer on biopsy as PSA (AUC for both, 0.64; Fig 3A), with a superior tumor specificity (0.98 *v* 0.81), considering both as continuous variables (Appendix Table A2). The prognostic value of epiCaPture was next studied. AUCs for detecting high-grade, high-risk D’Amico and CAPRA were 0.86, 0.83, and 0.80, respectively (Figs 3B-3D), with sensitivities of 0.85, 0.70, and 0.67 and NPV for all three prognostic end points greater than 0.94 (Appendix Table A2). Combining epiCaPture and PSA correctly predicted all high-grade cancers on biopsy (AUC, 0.95). Not forgetting the clinical significance of Gleason 7 disease, we also applied the revised five-grade grouping system,^18^ with a view to detecting group 3 (Gleason 4 and 3) and above. The combination of epiCaPture and PSA gave the best model (AUC, 0.82; sensitivity, 0.73; specificity, 0.76; Appendix Fig A1; Appendix Table A2).

We next evaluated the training set data to select an epiCaPture score cutoff that could best inform the decision on whether to perform a biopsy. Applying a threshold greater than 0.73 detected 38% of all cancers in the test set. Increasing this cutoff to greater than 1.25 detected 85% of high-grade disease, with a tumor specificity of 86% (Figs 3E-3H). Compared with the clinically accepted PSA threshold of 3 ng/mL, this represents a 75% improvement in tumor specificity. Within the total study cohort, 245 men fell within the prognostically challenging PSA 4 to 10 ng/mL bracket. Of these 245, 35 (14.29%) men had high-grade cancer. At the greater than 1.25 threshold, 14 of 35 (40%) of these men with high-grade disease tested positive for epiCaPture. Finally, DCA showed that at this threshold, epiCaPture demonstrated a net benefit over PSA in the decision on whether to perform a biopsy, enabling a 15% reduction in prostate biopsies (Figs 3I and 3J).

### epiCaPture Genes Show High Reproducibility by Independent Analysis

**Fig 4.**
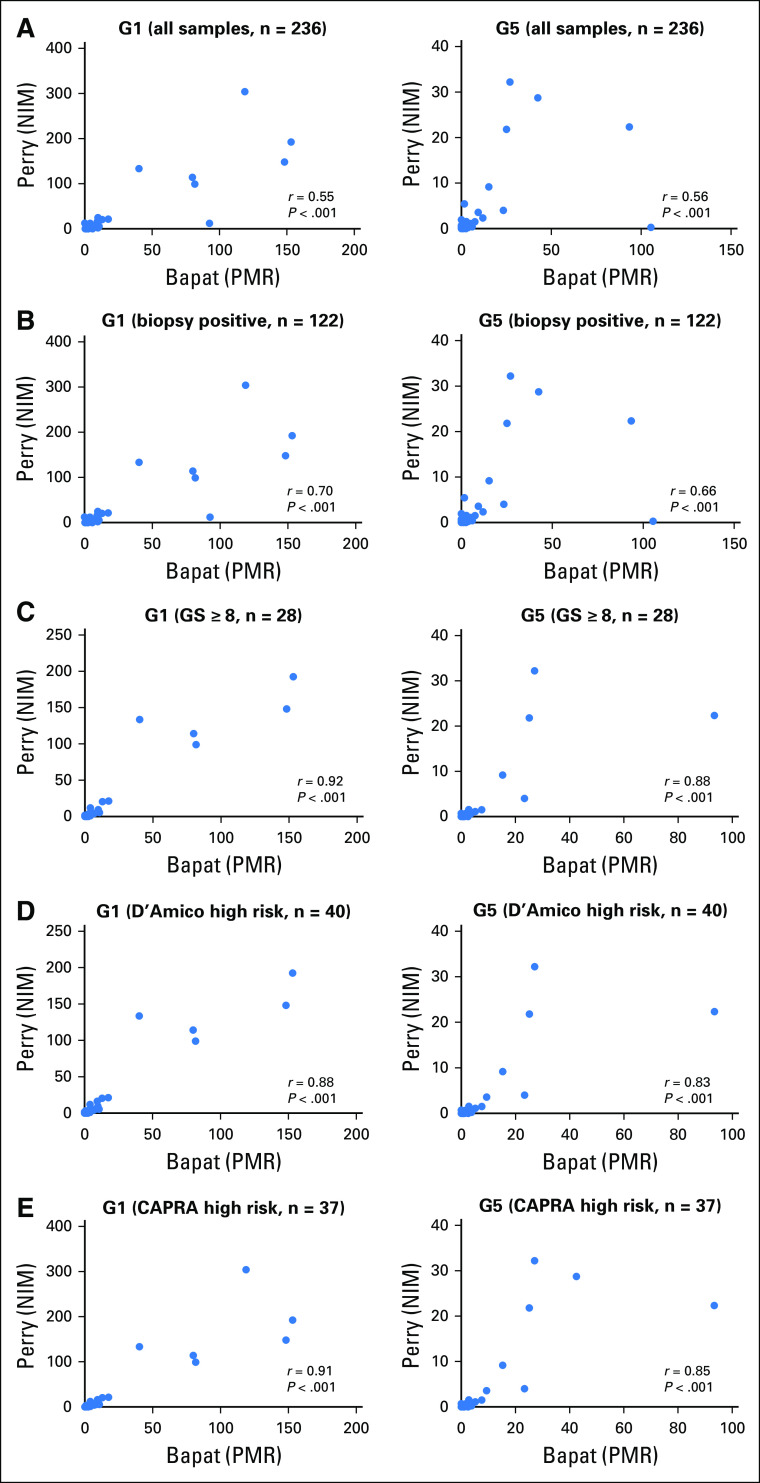
Robustness of Epigenetic Cancer of the Prostate Test in Urine (epiCaPture) assays by independent verification. (A) Two of the six epiCaPture assays (G1: *GSTP1* and G5: *APC*) were analyzed independently in laboratories in Ireland (Perry) and Canada (Bapat) by quantitative methylation specific PCR in urine sediments from 236 of 453 men in the study. Relevant subsets were then considered by applying the study end points: (B) men with biopsy-positive disease, (C) men with high-grade (Gleason score greater than or equal to 8) prostate cancer, and men with high-risk prostate cancer defined by (D) D’Amico, and (E) CAPRA (Cancer of the Prostate Risk Assessment) classification. Data from the two laboratories are differentiated by the labels Perry and Bapat to indicate the principle investigator at each site. In each data set, the normalized index of methylation (NIM) and percent methylated ratio (PMR) represent the normalized data value for each sample. Correlation was calculated using Spearman rank correlation.

To examine the robustness of epiCaPture, a matched quantitative methylation specific PCR analysis of two of the panel (G1: *GSTP1*, and G5: *APC*) was carried out in two independent laboratories on 236 of the urine samples. Although the genes were the same, some differences existed in the target and control amplicon sequences and post-PCR analytical formulae (Data Supplement). Considering the sample set as a whole, irrespective of grade or other factors, both genes demonstrated a moderate correlation in methylation values between the two laboratories (*r* = 0.55, *r* = 0.56; *P* < .001; Fig 4A). However, the correlation increased for both genes, when considering only the biopsy-positive samples (n = 122; *r* = 0.70, *r* = 0.66; *P* < .001; Fig 4B), the high-grade samples (*r* = 0.92, *r* = 0.88; *P* < .001; Fig 4C) or high-risk samples by applying either D’Amico (*r* = 0.88, *r* = 0.83; *P* < .001; Fig 4D), or CAPRA (*r* = 0.91, *r* = 0.85; *P* < .001; Fig 4E) risk stratification. Furthermore, matched data were available for 18 of 30 high-risk/grade samples that were epiCaPture negative, of which 15 (83.33%) and 16 (88.89%) were also negative for G1 and G5, respectively, in the independent analysis.

### Parallel epiCaPture Analysis in Urine and Matched Biopsy Cores Highlights Value of Liquid Biopsy

**Fig A2.**
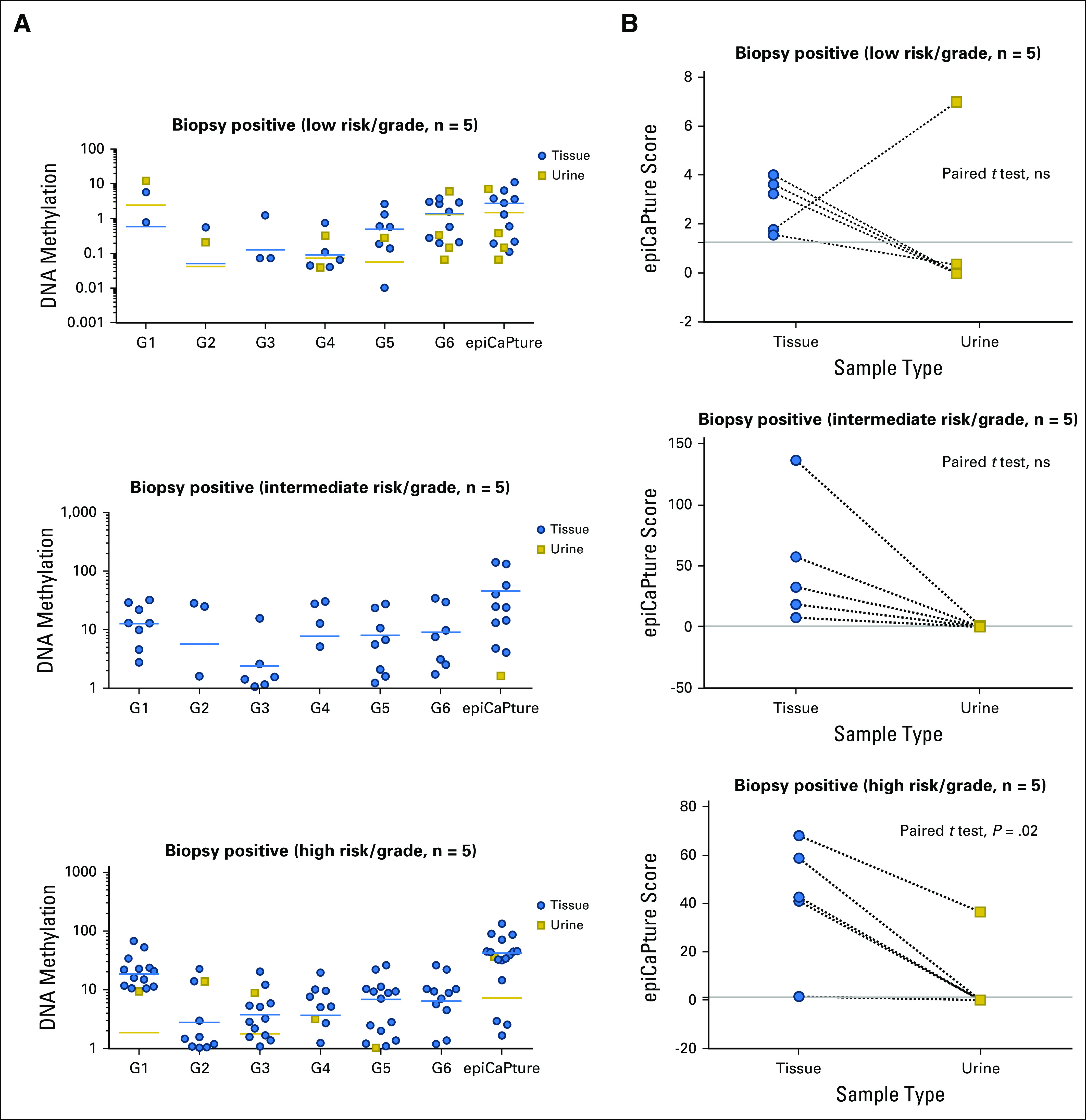
Parallel epiCaPture analysis in urine and matched biopsy cores. epiCaPture was performed in urine and matched formalin-fixed paraffin embedded biopsy cores from randomly selected biopsy-positive (n = 15: low-risk [CAPRA and D’Amico, Gleason score 6; intermediate risk, Gleason score 7; and high risk, Gleason score ≥ 8]) patients. (A) Parallel analysis of epiCaPture genes (G1-G6) in biopsy tissue cores and urine. DNA methylation of each gene is indicated by the NIM (normalised index of methylation), and for the collective panel, by the epiCaPture score (NIM sum G1-G6). Each point represents a single sample (biopsy core or urine), with the mean for each group indicated by a horizontal line (yellow, urine; blue, tissue). For ease of interpretation, DNA methylation is plotted on a logarithmic axis, and samples with no methylation are not shown. (B) Paired T tests on matched biopsy cores and urine reveal higher epiCaPture scores in tissues than in urine. For each patient, a single tissue epiCaPture score was calculated using the average across all cores studied (1-5). The epiCaPture threshold for urine positivity (1.25) is indicated by a gray line. ns, not significant.

PCa is renowned for its multifocal nature. A concern for any liquid biopsy is therefore how well it captures molecular aberrations occurring within discrete tumor foci. Matched formalin-fixed paraffin-embedded biopsy cores were available for a proportion of patients, enabling parallel epiCaPture analysis on multiple tumor foci and urine from individual men (n = 15). G1 to G6 methylation was higher in biopsy cores than in urine (Appendix Fig A2A), indicative of the higher tumor content in tissue over liquid biopsies. Similarly, individuals demonstrated higher epiCaPture scores in tissues than in urine (Appendix Fig A2B). The most interesting insights came from the matched analysis of urine with multiple biopsy cores, of which two notable cases are highlighted.

**Fig 5.**
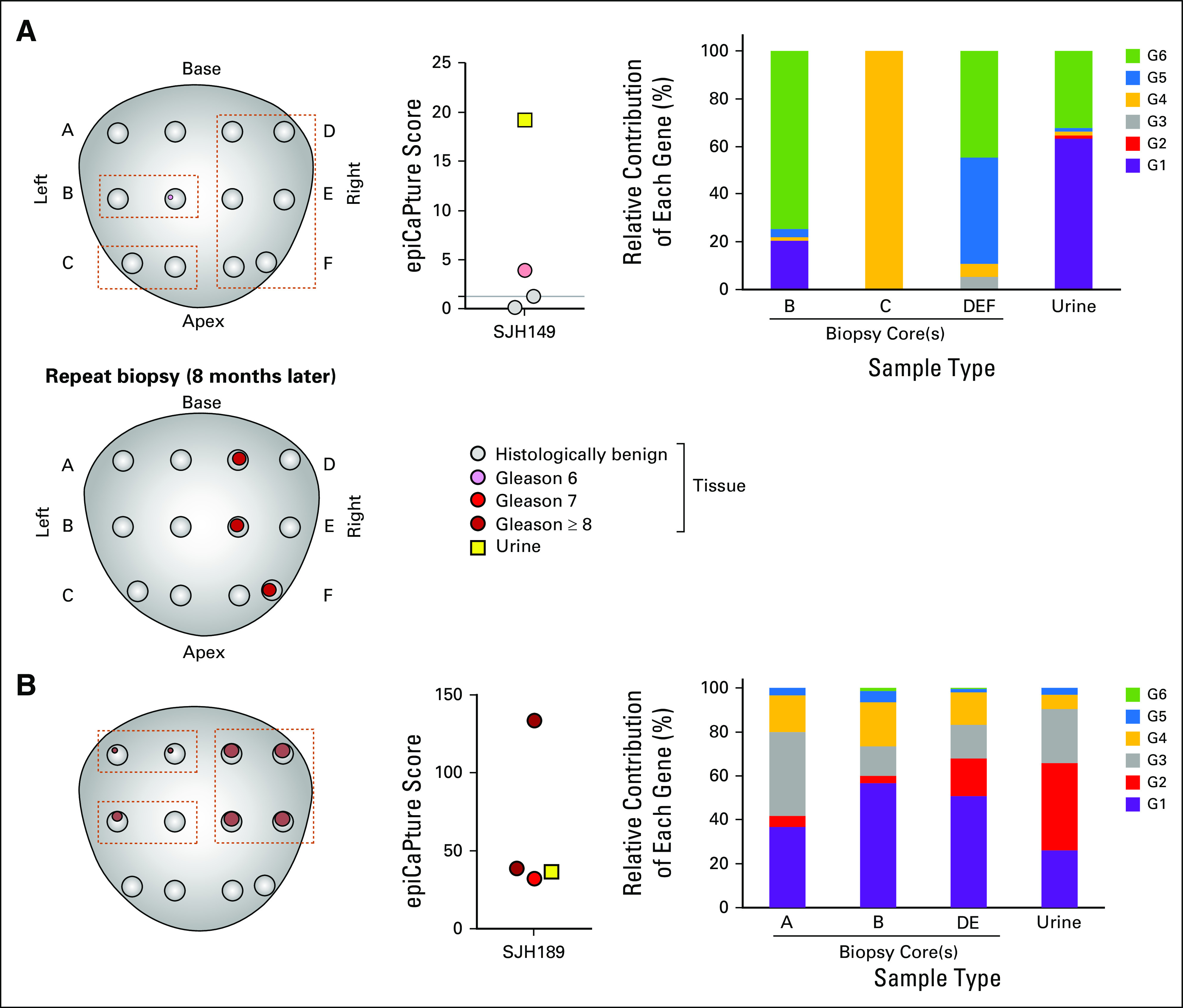
Parallel Epigenetic Cancer of the Prostate Test in Urine (epiCaPture) analysis in liquid and tissue biopsies. Prostate maps are for indicative purposes only and are not drawn to scale. A standard 12-core biopsy is illustrated. In some cases, more than 12 cores were taken. Tumor presence is denoted by pink (Gleason score 6), red (Gleason score 7), or brown (Gleason score greater than or equal to 8) circles, with size indicative of percentage tumor in each core. Dashed brown lines indicate cores that were used/combined for DNA extraction. epiCaPture analysis was performed in parallel on matched biopsy cores (circles) and prebiopsy urine (yellow square). The threshold for positivity (epiCaPture score greater than 1.25) is indicated on the graph. epiCaPture fingerprints indicate the relative contribution of each of the six genes to the epiCaPture score calculated for matched tissue cores and urine. (A) Low-risk prostate cancer SJH149 (62 years of age; prostate-specific antigen, 3.08 ng/mL) demonstrated that urine as a liquid biopsy is a viable alternative to sampling bias inherent to tissue needle biopsies. Original biopsy: tumor occupying less than 1% total volume in one of two cores on the left-hand side. DNA was extracted from cores B, C, and D + E + F. Repeat biopsy: tumor was present in the medial, anterior, and posterior of the right-hand side of the gland. (B) High-risk prostate cancer SJH189 (80 years of age; prostate-specific antigen, 6.63 ng/mL) demonstrated similar epiCaPture fingerprint across different tumor areas sampled on biopsy and in urine.

Patient SJH149 was diagnosed with a minute focus of Gleason score 6 adenocarcinoma. His urine epiCaPture score was strongly positive. A repeat biopsy revealed high-grade cancer in 40% of cores on the right side (Fig 5A). epiCaPture analysis of the original biopsy cores revealed DNA methylation, albeit at a magnitude lower than detected in urine. Notably, the epiCaPture fingerprint (ie, the relative contribution of each gene to the epiCaPture score) differed between tissue cores and urine. This finding could suggest that the source of the tumor DNA analyzed in urine could have been the high-grade tumor, which was not sampled on the original biopsy.

In contrast, patient SJH189 was diagnosed with a high-percentage, high-grade (intermixed patterns of grade 4 and 5) PCa on both sides of his prostate. His urine epiCaPture score was strongly positive, as were his tissue cores (Fig 5B). His epiCaPture fingerprint was comparable across urine and tissue cores, with a strong contribution from G1 to G4, smaller contribution from G5, and G6 negligible. So, despite pathologic heterogeneity within the gland, the three tumor areas seemed to be epigenetically homogeneous.

## DISCUSSION

In this urine PCa biomarker study, we evaluated the performance of epiCaPture, a six-gene DNA methylation panel, for noninvasive detection of PCa and more specifically high-grade or high-risk PCa in urine from patients referred for prostate biopsy on the basis of elevated PSA and/or abnormal DRE. Complying with REMARK guidelines, we describe a new DNA methylation-based urine test for early detection of aggressive PCa.

Many prostate tumors display a protracted disease trajectory, almost indolent in behavior.^19^ The widespread use of the serum PSA test in many countries has significantly increased the incidence and thus overtreatment of these low-risk cancers with little likelihood of clinical manifestation.^20-23^ An important first consideration in developing epiCaPture was to therefore critically appraise the evidence for prognostic utility of each constituent gene. Using two independent cohorts of prostate tissues, we showed that the DNA methylation of epiCaPture genes increases with aggressiveness. Conversely, however, it is estimated that approximately two thirds of men undergoing invasive prostate biopsy have no tumor diagnosed, because of the high false-positive rate of serum PSA.^23,24^ As such, millions of unnecessary biopsies are performed worldwide each year, at great cost to our health care systems and causing significant anxiety, trauma, and comorbidities for patients.^25,26^ PSA accuracy has been improved by the development of algorithms incorporating different PSA isoforms, namely the four-kallikrein and Prostate Health Index tests, with AUC values in the region of 0.7 (all PCa) and 0.71 to 0.79 (high-grade PCa, Gleason score greater than or equal to 7).^4,27,28^ However, PSA is a single biomarker; used alone it does not address PCa heterogeneity or inform on disease biology.

The multifocal nature of PCa together with small sampling (less than 5%) of the gland by TRUS biopsy is problematic; approximately 20% of tumors are upgraded on radical prostatectomy because of sampling error.^29^ Although sophisticated imaging technologies, such as magnetic resonance imaging, may improve the accuracy of prostate biopsy,^30^ their widespread application is hampered by high cost, access to specialist equipment and personnel, interobserver subjectivity, and requirement for anesthesia. Alternatively, transperineal biopsy enables a more complete sampling of the gland, including the anterior prostate, but also requires anesthesia and is thus unsuitable as a population-based detection method for such a prevalent disease.

The dearth of blood-based prognostic biomarkers as a screening tool for aggressive PCa in conjunction with limitations of the prostate biopsy in its various forms has led to excitement around urine biomarkers. Physical manipulation of the prostate by DRE, followed by a first-void urinalysis, could provide a simple, holistic insight into molecular alterations occurring in the prostate gland. Indeed, many studies have demonstrated significant value in detecting and noninvasively monitoring several genitourinary malignancies through urine.^31-34^ Some other prognostic urine indicators of aggressive PCa have already been described. For example, the Mi Prostate Score (MiPS), which incorporates the prostate-specific *TMPRSS2-ERG* fusion and *PCA3* transcripts in urine, in conjunction with serum PSA, delivers AUCs of 0.75 and 0.78 for detecting all PCa and high-grade PCa, respectively.^35^ However, the prognostic value of this combination has been questioned by others.^36,37^ Combining expression of three genes (*HOXC6*, *TRD1*, and *DLX1*) functionally implicated in PCa with serum PSA detected clinically significant disease (Gleason score greater than or equal to 7 on biopsy), with an AUC of 0.81.^38^

Epigenetic alterations represent a particularly attractive source of biomarkers: promoter hypermethylation of many regulatory genes occurs early during prostate cancer initiation, it is often prognostic, and assessment of DNA is a more robust analyte than RNA. Early studies demonstrated detection of *GSTP1* methylation in urine from patients with PCa, although sensitivity was poor (less than 30%).^39^ Follow-up work expanded the repertoire of markers (*GSTP1*, *APC*, and *RARβ2*), which improved sensitivity to approximately 60%.^40^ The epiCaPture panel represents five commonly perturbed pathways in PCa: intracellular detoxification, Wnt and IGF axes, cell cycle, and prostaglandin biosynthesis. Assessing its diagnostic performance in terms of ability to predict cancer (irrespective of grade) on biopsy, epiCaPture was equivalent to PSA, with AUCs of 0.64. However, the reality of clinical practice is that PSA is not used as a continuous variable, with a threshold applied to guide decisions on whether to biopsy. Taking the widely used cut-off of 3 ng/mL, the performance of PSA decreased to AUC of 0.55, in line with previous reports. In contrast, applying an epiCaPture threshold (greater than 0.73; developed on training set) did not markedly alter its diagnostic performance (AUC, 0.68) and delivered a 79% improvement over the tumor specificity of PSA.

In an effort to address the prognostic utility of epiCaPture within the context of the clinically heterogeneous Gleason 7 disease, we applied the revised five-grade grouping system,^18^ in addition to two widely used risk-classification systems. It should be noted all men with aggressive cancers in this study had an elevated PSA; however, its specificity was poor. The epiCaPture performance characteristics represent a potential improvement on existing methods for patient stratification and for determining the need to perform biopsy, detecting 85% of high-grade tumors, with an NPV of 98%. Furthermore, the overlap of *GSTP1* and *APC* in the two GAP1 urine DNA methylation biomarker panels revealed excellent correlation in detection of aggressive PCa, illustrating the robustness of these biomarkers. In the context of the diagnostic gray zone, PSA 4 to 10 ng/mL, epiCaPture demonstrated potential for further classifying patients, positive in 40% of men with high-grade PCa. Applying DCA with epiCaPture showed a net reduction of approximately 15% in prostate biopsies. Given the high prevalence of this disease, this has the potential to dramatically reduce the number of biopsies being performed. Although these data are promising, a large prospective clinical trial is needed to assess whether epiCaPture offers a survival advantage.

A limitation of our study is that the cohort is young; analysis was only possible using biopsy as the end point. We cannot rule out the possibility that a proportion of our epiCaPture-positive urine samples in men with low-grade or benign tissues were indeed tumor cases missed on biopsy. Longer-term follow-up is thus needed to more accurately assess the false-positive rate of epiCaPture (calculated at 5% in this study, but presumed lower because of sampling error of TRUS biopsy). Evidence actually shows that DNA methylation detected in histologically benign tissue (due to an epigenetic field effect from the cancer), can be used as a tool to rule out the possibility that the biopsy missed the tumor. For example, the Confirm MDx test, which measures DNA methylation at three genes (*GSTP1*, *APC*, and *RASSF1*) in histologically benign biopsy tissue cores, is used to guide the decision to repeat a biopsy.^41^

Conversely, 14 of 47 (29.79%) high-grade tumors were epiCaPture-negative. Independent analysis revealed concordant negativity in more than 80% of these men, and lowering the epiCaPture threshold did alter this result; they were negative across all six markers. Possible explanations such as low tumor volume were excluded. We believe that the most likely reason is interoperator differences in DRE; these high-risk/grade cancers were epiCaPture negative because they had insufficient/undetectable prostate cells present in their urine. Future work will focus on incorporating expression of a prostate-specific marker (ie, *KLK2* or *KLK3*) into epiCaPture, to enable confirmation of the presence of prostate cells in the urine, validating its suitability for epiCaPture analysis and ultimately improving the performance of the test. Future work will also investigate the intra-individual reproducibility of epiCaPture by repeated measures in men.

Finally, to the best of our knowledge, this is the first PCa biomarker study to perform a matched analysis of tissue and liquid biopsies. A more extensive project is now warranted to fully assess the effects of the multifocal nature of PCa on signatures derived from liquid biopsies.

epiCaPture could be used in conjunction with existing tools (ie, PSA and risk calculators) to help guide the decision to biopsy. On further validation and cost-benefit analysis, epiCaPture may have utility as a population-based stratification tool to identify those men who require a biopsy, to alleviate patient harms and hospital burdens of over-performing biopsies on patients.

## Figures and Tables

**Table A1.**
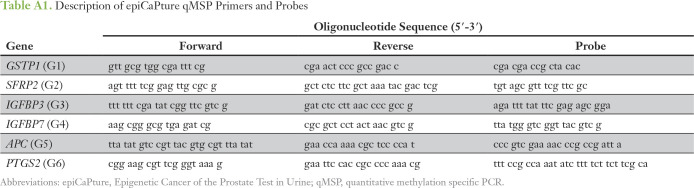
Description of epiCaPture qMSP Primers and Probes

**Table A2.**
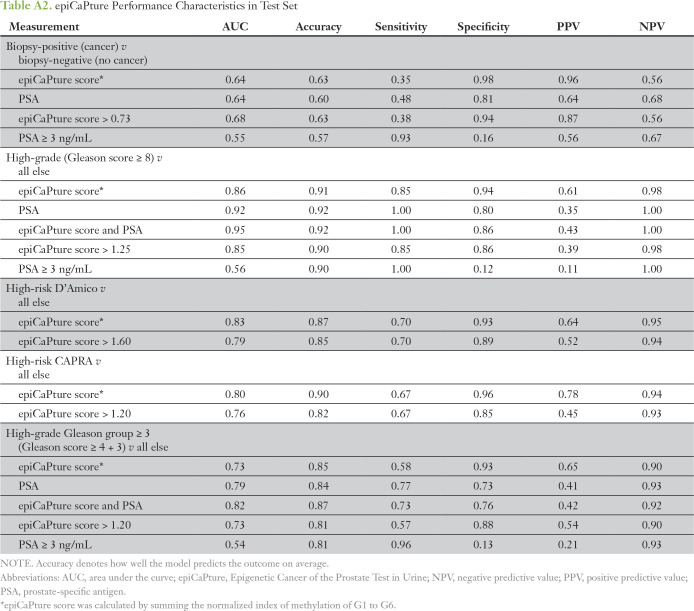
epiCaPture Performance Characteristics in Test Set
